# Development of a Machine Learning Model for Classifying Cooking Recipes According to Dietary Styles

**DOI:** 10.3390/foods13050667

**Published:** 2024-02-22

**Authors:** Miwa Yamaguchi, Michihiro Araki, Kazuki Hamada, Tetsuya Nojiri, Nobuo Nishi

**Affiliations:** 1 National Institute of Health and Nutrition, National Institutes of Biomedical Innovation, Health and Nutrition, Osaka 566-0002, Japan; araki@nibiohn.go.jp (M.A.); nishi.nobuo.24@slcn.ac.jp (N.N.); 2 Oishi Kenko Inc., Tokyo 103-0024, Japan; manemone@oishi-kenko.com (K.H.); tetsuya@oishi-kenko.com (T.N.); 3 Graduate School of Public Health, St. Luke’s International University, Tokyo 104-0045, Japan

**Keywords:** prediction model, Japanese diet, Chinese diet, Western diet

## Abstract

To complement classical methods for identifying Japanese, Chinese, and Western dietary styles, this study aimed to develop a machine learning model. This study utilized 604 features from 8183 cooking recipes based on a Japanese recipe site. The data were randomly divided into training, validation, and test sets for each dietary style at a 60:20:20 ratio. Six machine learning models were developed in this study to effectively classify cooking recipes according to dietary styles. The evaluation indicators were above 0.8 for all models in each dietary style. The top ten features were extracted from each model, and the features common to three or more models were employed as the best predictive features. Five well-predicted features were indicated for the following seasonings: soy sauce, miso (fermented soy beans), and mirin (sweet cooking rice wine) in the Japanese diet; oyster sauce and doubanjiang (chili bean sauce) in the Chinese diet; and olive oil in the Western diet. Predictions by broth were indicated in each diet, such as dashi in the Japanese diet, chicken soup in the Chinese diet, and consommé in the Western diet. The prediction model suggested that seasonings and broths could be used to predict dietary styles.

## 1. Introduction

The basic combination of traditional Japanese diets, known as washoku in Japanese, consists of cooked rice with one soup and three side dishes that make diets low-fat, low-energy, and well-balanced [1]. “Washoku, the traditional dietary cultures of the Japanese” was inscribed in UNESCO’s Representative List of the Intangible Cultural Heritage of Humanity in 2013 [2]. After the Great Kanto Earthquake of 1923, Chinese and Western cuisines have disseminated across the entire Japanese population, modifying various dishes into unique Japanese versions [3]. Today, Chinese and Western diets are familiar to the Japanese people in addition to traditional Japanese diet. According to the database of the National Health and Nutrition Survey, Japan, from 2003 to 2015, a study indicated a decrease in the dietary pattern of “plant food and fish,” which is usually classified in traditional diets, and an increase in the dietary pattern of “bread and dairy” and “animal food and oil”, which are usually included in the Western diet, suggesting continuous Westernization [4].

A systematic review of the Japanese diet indicated that the top three applicable categories were soy beans/soy bean-derived products, seafood, and vegetables, followed by rice and miso soup [5]. Miso is a paste made from molded rice, cooked soy beans, and salt [6]. Miso soup is composed of miso and Japanese broth, known as “dashi” in Japanese, which is usually made from kelp and dried bonito [7]. From the 1970s to the 1980s in Japan, fried Chinese noodles and dumplings from the Chinese diet and sandwiches, spaghetti, hamburgers, toasts, and cream stews from Western diets were gradually consumed as daily dishes [3].

The Japanese diet has been reported as one of the factors responsible for the longevity of Japanese people [8]. However, it is not known whether the traditional Japanese diet is superior to Japanese–Chinese and Japanese–Western diets in relation to longevity. Several studies have examined the relationships between dietary patterns and health-related indicators, including cancer [9], cardiovascular disease [10], and dementia [11]. There are several classical methods for identifying dietary patterns such as dietary quality scores, principal component analysis, factor analysis, clustering analysis, and reduced-rank regression [12]. A systematic review was previously conducted to examine the reproducibility of dietary patterns using principal component analysis [13]. The review reported that some major dietary patterns are relatively reproducible, but others are not found in different populations within a country. The interpretation of dietary styles should be carefully considered because the dietary styles for traditional methods were defined in each study.

Machine learning algorithms have recently been used in different areas of nutrition to complement current dietary pattern analyses, which may not integrate sufficient dietary variation [14]. Classifying pictures of food into categories is one way that machine learning could become a useful complementary method for improving the precision and validity of dietary measurements [14]. The systematic review reported that supervised learning algorithms were mostly used to assess food intake using a food frequency questionnaire [15]. The review selected 36 studies, out of which 23 used a classification algorithm. One of the studies used machine learning algorithms to predict a healthy diet based on food intake [16]. Another study clarified the specific food groups that can predict and classify adults with obesity and/or diabetes [17]. Yu et al. [18] used machine learning algorithms to determine food groups related to the incidence of bladder cancer. These previous reports demonstrate that text-based information related to dishes, such as cooking recipes, may also be applied to evaluate dietary styles.

Current Japanese dietary styles are diverse and challenging to classify. Even nutrition specialists, such as dieticians, do not have a standard for defining dietary styles. One review reported the difficulty in defining the Japanese diet because consistent definitions have not been established [19]. The present study refers to traditional Japanese, Japanese–Chinese, and Japanese–Western diets as Japanese, Chinese, and Western diets for good legibility. It is necessary to develop a complementary tool for classical methods to identify evidence-based dietary styles. Such a prediction model would support researchers in properly naming dietary patterns resulting from classical methods. Moreover, such a study can contribute to preserving the Japanese dietary style by identifying the understandable characteristics of this diet. Therefore, this study aimed to develop a machine learning model for classifying cooking recipes into Japanese, Chinese, and Western dietary styles in Japan.

## 2. Materials and Methods

### 2.1. Database

To build a dataset for the binary classification task in each dietary style, 9092 cooking recipes were collected from the “Oishi Kenko” app, supporting healthy dietary habits [20]. Among these, 909 recipes characterized by two or more dietary styles in one recipe were excluded, leaving 8183 cooking recipes representing Japanese, Chinese, or Western diets. The recipe examples can be found in Table S1. Each recipe’s dietary style was determined by two registered dieticians from a pool of ten within the company. Dietary style was classified by prioritizing dish name, photos, seasonings, and ingredients. The dieticians made comprehensive judgments considering annotation data and consistency with other recipes to assign the dietary style. In total, 27 annotations were utilized to characterize the recipes (Table 1), falling into four types. The first type covered various recipe characteristics (e.g., cooking type, cooking genre, main ingredients, arrangement type, main seasoning type, situation, suitable event, and basic or arrangement). The second focused on taste, flavor, and nutrients (e.g., taste characteristics, texture, nutrition point, smell characteristics, and nutritional value). The third outlined cooking methodologies (e.g., finishing cooking method, temperature, suitable time zone, estimated cooking time, season, easy point, necessary cooking utensils, and material). The fourth addressed considerations for individuals with health issues or dietary restrictions (e.g., infectious disease countermeasures, effects on the digestive system, trouble symptoms, cooking difficulty, and allergen-free). Nutritional and ingredient data were sourced from the Standard Tables of Food Composition in Japan 2015 (Seventh Revised Edition) [21], comprising 12 nutrients (e.g., energy, macronutrients, and micronutrients) and 19 ingredients (e.g., vegetables, fruits, and meat). Examples of these features are detailed in Table 1 for each dataset component. A total of 1547 explanatory features were initially processed, including 366 annotations, 50 nutrients, and 1131 ingredients. Following the exclusion of unavailable features, the final analysis included 604 features, which underwent one-hot encoding to convert categorical variables.

### 2.2. Statistical Analysis

**Figure 1:**
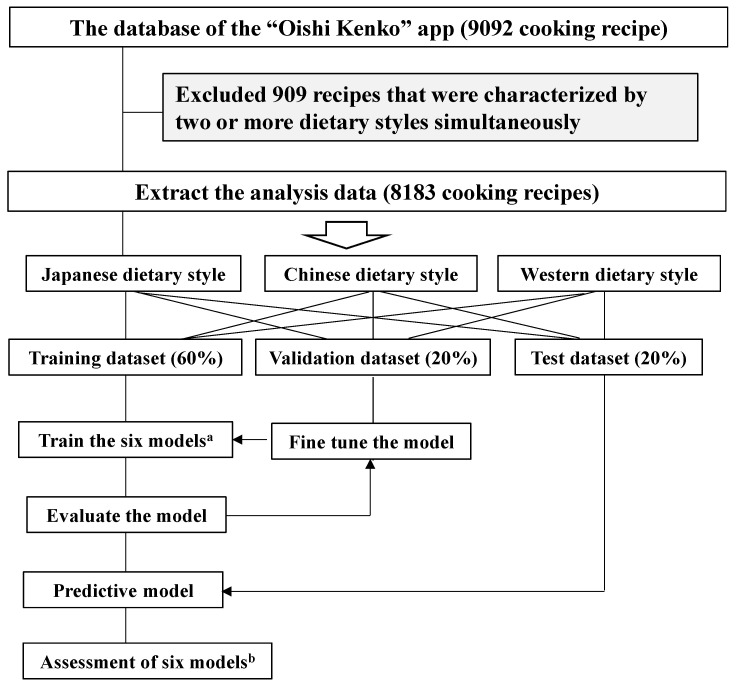
Flow chart of data analyses. ^a^ The following six models were used: a random forest classifier, logistic regression, support vector classifier, extreme gradient boosting, light gradient boosting machine, and deep neural network. ^b^ The six models were assessed using four indices: accuracy, area under the receiver operating characteristic curve, F1-score, and Matthew’s correlation coefficient.

The data were randomly divided into training data (60%), validation data (20%), and test data (20%) for each dietary style (Japanese, Chinese, and Western), maintaining the ratio of positive to negative data. The flow chart depicting the analyses is shown in Figure 1. We fine-tuned the parameters and trained the model to prevent overfitting and underfitting. Additionally, we assessed the model’s performance using test data that were not part of the model training process to ensure appropriate performance.

To extract important features that are both robust and specific to each machine learning algorithm, this study selected six machine learning models to which the Shapley additive explanations (SHAP) algorithm [22] can be applied and that can run calculations on the computer this study used. The following six machine learning models have been developed: a random forest classifier (RFC) [23], logistic regression (LR), support vector classifier (SVC) [24], extreme gradient boosting (XGB) [25], light gradient boosting machine (LGBM) [26], and deep neural network (DNN) [27]. The 4-fold cross-validation was suitable for evaluating the accuracy of the six learning models. Therefore, the hyperparameters of the model were determined by 4-fold cross-validation of the training data and a grid search. For data processing, the explanatory variables were standardized using means and standard deviations. The models were evaluated using four indices: accuracy (ACC), area under the receiver operating characteristic curve (AUC), F1-score, and Matthew’s correlation coefficient (MCC). The confusion matrix was constructed before performing calculations using the six models. The ACC was used to correctly assess the ability to differentiate between positive and negative results [28]. The equation is below:

True positive (TP) =the number of cases correctly identified as positive

False positive (FP) =the number of cases incorrectly identified as positive

$$True\;negative\;\left(TN\right)\;=the\;number\;of\;cases\;correctly\;identified\;as\;negative$$

False negative (FN) = the number of cases incorrectly identified as negative

$$Accuracy\;=\;\frac{TP\;+TN}{TP\;+\;TN\;+\;FP\;+FN}$$

The AUC was used to assess the classification performance of each model. AUC is the area under the receiver operating characteristic (ROC) curve. The x-axis in the ROC curve indicates $the\;false\;positive\;rate\;\left(1-specificity\right)$, and the y-axis indicates the true positive rate (sensitivity) [29].

$$False\;positive\;rate\;\left(1-specificity\right)\;=\;\frac{FP}{FP\;+\;TN}$$

$$True\;positive\;rate\;(sensitivity)\;=\frac{TP}{TP\;+\;FN}$$

An AUC value close to 1 indicates better binary classification. The closer the ROC curve is to the upper-left corner of the graph, the higher the accuracy of the test, because in the upper-left corner, the false positive rate = 0 and the true positive rate = 1.

The F1-score (range, 0–1) is defined as the harmonic mean of precision and recall, which has a trade-off relationship.

$$F1-score\;=\;\frac{2TP}{2TP\;+\;FP\;+\;FN}=\frac{2\;(precision\;\times \;recall)}{precision\;+\;recall}$$

The minimum F1-score is reached for TP = 0 when all positive samples are misclassified. The maximum F1-score is reached for FP = FN = 0 when it is a perfect classification.

MCC is a special case of the ∅(phi) coefficient [30] for 2 × 2 confusion matrices.

$$MCC=\frac{\left(TP\;\times \;TN)\;-\;(FP\;\times \;FN\right)}{\sqrt{\left(TP\;+\;FP)\;\times \;(TP\;+\;FN)\;\times \;(TN\;+\;FP)\;\times \;(TN\;+\;FN\right)}}$$

An MCC close to +1 indicates perfect classification for all other confusion matrix metrics, and −1 means the worst prediction, where all negative samples are predicted as positive, and vice versa [31].

The SHAP algorithm was applied to each model to calculate the correlation coefficient and identify the importance of each explanatory variable and its impact on the prediction [22]. A correlation analysis was not successfully performed in the SVC model because the model exhibited low reproducibility between the feature analysis and correlation. Important features were extracted for each dietary style as follows based on the calculated results: the top ten features were extracted from each model, and features common to half (i.e., three) or more of the models were used as well-predicted features to summarize the characteristics of the obtained results. The applicability of these models was confirmed in a previous study [32]. Python was used for the statistical analyses.

## 3. Results

Table 2 presents the evaluation of the six machine learning models used to classify cooking recipes into three dietary styles. The confusion matrix results of each dietary style are presented in the Supplementary Materials (Figures S1–S3). Accuracy, AUC, and F1-score exceeded 0.8 for all dietary types and models. The model with the highest average among the four evaluation indices for the six models was identified as the best model. The top performing models for each dietary type were LGBM for the Japanese diet, RFC for the Chinese diet, and DNN for the Western diet.

The ROC curves for all Japanese, Chinese, and Western dietary styles exhibited a trend toward the upper left, denoting high performance (Figures S4–S6). Similar trends were observed for the ROC curves of Japanese and Chinese dietary styles. For the Chinese dietary styles, the ROC curves of RFC, XGB, and LGBM were more prominently situated compared with those of other models, aligning with the trend of AUC scores.

Among the top ten features in the six models, five well-predicted features are highlighted in bold font in the Japanese diet (Table 3), Chinese diet (Table 4), and Western diet (Table 5). Three dietary styles exhibited positive correlations with specific seasonings: soy sauce, miso (fermented soy beans), and mirin (sweet-cooked rice wine) in the Japanese diet; oyster sauce and doubanjiang (chili bean sauce) in the Chinese diet; and olive oil in the Western diet. Broths emerged as strong predictors for each dietary style: dashi (and the flavor) for the Japanese diet, chicken broth for the Chinese diet, and consommé for the Western diet. Certain foods also predicted dietary styles: starch for the Chinese diet and dairy products, tomato, and garlic for the Western diet. Among the five items that predicted dietary styles, iodine was the only nutrient found in the Japanese diet.

Soy sauce in the Japanese diet appeared in five models, excluding the SVC model. In the Chinese diet, sesame oil, chicken broth, and oyster sauce were well-predicted features across all the six models. For the Western diet, olive oil was present in all the six models, whereas dairy products appeared in most models except the SVC model.

## 4. Discussion

This study developed a machine learning model to classify Japanese, Chinese, and Western dietary styles based on cooking recipe data, suggesting that seasonings and broths effectively differentiate between these dietary styles. To the best of our knowledge, this is the first study demonstrating the use of a machine learning model based on text features for identifying the three national dietary styles in Japan.

Six major dietary patterns, including Japanese and Western patterns, were identified in a systematic review analyzing 65 articles on national dietary patterns using the principal component procedure [13]. The Japanese pattern was characterized by higher intakes of mushrooms, seaweeds, potatoes, vegetables, pickles, pulses, seasonings, fruits, and fish and shellfish [13]. This study did not highlight these ingredients as the best practice features in the Japanese dietary pattern. However, a notable finding in our study is that only iodine in the Japanese diet was presented as a nutrient among the top five components of the three dietary styles. Iodine may reflect the use of seaweed and seafood in the Japanese diet [33]. The inclusion of seaweeds, fish, and shellfish in our results aligns with the findings in the review [13]. While the review mentioned seasoning as a characteristic of the Japanese diet, it did not provide detailed information on the type of seasoning [13]. The present study revealed that soy sauce was frequently presented as a well-predicted seasoning feature in the Japanese diet, making it easily associated with Japanese cuisine.

Interestingly, previous studies using dietary patterns did not identify a distinct Chinese dietary pattern [13]. The naming of each dietary pattern is usually based on the author’s perception during a principal component analysis [12]. The low significance of the author’s perception for distinguishing between Japanese and Chinese styles may be due to the similarity in ingredients and seasonings within these countries. However, our study identified robust features such as sesame oil, chicken broth, and oyster sauce in the Chinese diet. These tastes and flavors contribute to the identification of the Chinese diet. Additionally, our study revealed starch as a feature in the Chinese diet, with cornstarch (i.e., corn flour) commonly used in Chinese cooking for thickening soup and quick frying with corn flour [34]. Recognizing the classification of Chinese diets is essential, particularly if these characteristics are associated with non-communicable diseases. In a Chinese meta-analysis, the traditional Chinese dietary pattern, including starchy foods (i.e., rice, wheat, and tubers), vegetables, and high-protein foods (i.e., pork) was associated with a lower risk of overweight/obesity [35]. Although Chinese dietary styles have been adapted in Japan, the presence of Chinese diets within Japanese food culture should be acknowledged.

In the Western diet, olive oil was present in all six models, while dairy products appeared in most models in this study, except the SVC model. A high intake of olive oil and moderate intake of dairy products are associated with the Mediterranean diet, known for reducing the risk of cardiovascular disease and cancer and enhancing cognitive health [36]. Notably, the well-predicted features in the Western diet in this study included ingredients such as dairy products, tomatoes, and garlic. These items might contribute to the foundational taste of the Western diet owing to their glutamic acid content [37].

Unlike in a previous review [4], this study did not highlight protein-sourced foods as significant features in the Western diet. This previous review investigated 13-year trends in dietary patterns among Japanese adults aged over 20 years and revealed an increasing trend in the “animal food and oil” pattern, characterized by higher consumption of red and processed meat, eggs, vegetable oil, and other vegetables across most generations [4]. However, the recipe database used in this study prioritized healthy diets, and hence, red meat (such as beef and processed meat) was not frequently featured in the recipes.

This study identified the best model for each dietary style among the six models based on accuracy, AUC, F1-score, and MCC. While the SVC model proved effective in predicting features for each dietary style, it lacked a correlation analysis owing to low reproducibility between the feature analysis and correlation. Additionally, the best model (DNN) for the Western diet did not include consommé. Implementing ensemble methods combining results from several models can enhance the predictive performance [38]. Therefore, it is important to assess the comprehensive results by utilizing not just one (e.g., SVC or DNN models) but several suitable models.

The strength of this study lies in the extraction of explicit knowledge using a machine learning model from the implicit knowledge inherent in nutrition specialists’ dietary style classifications. However, several notable limitations exist. First, the feasibility of other databases remains unclear as this study relied on only one company’s database [8]. In Japan, various types of Japanese, Chinese, and Western dietary styles exist other than those used in the present data. More data sources should be introduced to demonstrate the robustness of the findings in the future. In addition, the dietary style of recipes used for the training data was determined by only two registered dieticians. Second, while the present model can generally identify dietary style characteristics, some aspects of its generalizability might be limited because the considered recipes focused on health considerations determined by dieticians. Third, this study excluded various cooking recipe types such as Korean and ethnic recipes, as well as their combinations with Japanese, Chinese, and Western diets. The current model focused solely on classifying cooking recipes into three major dietary styles, presenting a challenge for future studies aiming to accommodate diverse dietary styles.

## 5. Conclusions

This study developed a machine learning model that classifies cooking recipes into Japanese, Chinese, and Western dietary styles using a recipe database, indicating that seasonings and broths can effectively aid in such classifications. This study also proposed a complementary tool to investigate the dietary patterns within the Japanese population alongside classical methods. The evidence-based classification of dietary styles complemented by the prediction model contributes to clarifying the relationship between dietary styles and health.

## Figures and Tables

**Table 1:** Features of the database.

Data	Components	Examples	n
Annotation data	Cooking type	Staple food, side dish, main dish	12
	Cooking genre	Korean food, ethnic food, others	6
	Finishing cooking method	Fry, bake, steam	9
	Main ingredients	Meat, vegetables, milk and dairy products	26
	Arrangement type	Calcium fortification, diets for morning sickness, Dysphagia diet	16
	Main seasoning type	Consommé, sweetener (sugar, mirin, honey), miso, sauce (Worcestershire sauce), other seasoning, dashi	15
	Taste characteristics	Dashi flavor, sesame flavor, soy sauce taste, salty	14
	Texture	No stimulation to oral cavity, easy to swallow, thicken	15
	Temperature	Room temperature, hot, very cold	5
	Suitable time zone	Anytime, for lunch, for breakfast	5
	Estimated cooking time	Within 5 min, within 15 min, within one hour cooking	7
	Season	Throughout a year, spring, summer	5
	Easy point	Only toaster oven, easy cooking, few cooking steps	25
	Nutrition point	Salt-free, “Diet” in the title, “Healthy” in the title	12
	Smell characteristics	No protein smell, easy to detect the smell of the ingredients	2
	Situation	For lunch box, for party, easy one-item lunch	7
	Necessary cooking utensils	Wooden pestle, oven, food processor	15
	Infectious disease countermeasures	Avoid infections, need caution about listeria food poisoning, need caution about the growth of bacteria	3
	Suitable event	New year (Osechi: traditional Japanese diets), Christmas, Valentine’s Day	6
	Material	Include dairy products, include tomato, include garlic, include potato, include green onion	80
	Nutrition value	Include foods with no measurement of potassium, include caffeine, very low fat and/or energy percent	34
	Effects on the digestive system	Good for digestion, adjust the intestinal environment, less likely to generate intestinal gas	2
	Basic or arrangement	Basic, arrangement	2
	Trouble symptoms	Complementary food for nutrition supply, abdominal distension, less force required for arms or hands	31
	Cooking method	Including frying process	1
	Cooking difficulty	Beginner, intermediate, advanced	4
	Allergen-free	Allergen-free of pork, allergen-free of sesame, allergen-free of apple	7
Nutrients (unit/dish) ^†^	Energy	Energy (kcal)	1
	Protein	Protein (g)	1
	Amino acid	Amino acid composition (g)	1
	Fat	Fat (g), triacylglycerol (g)	2
	Fatty acid	Saturated fatty acid (g), monounsaturated fatty acid (g), polyunsaturated fatty acid (mg)	3
	Cholesterol	Cholesterol (g)	1
	Carbohydrate	Carbohydrate (g), available carbohydrate (g)	2
	Fiber	Total fiber (mg), soluble fiber (g), insoluble fiber (g)	3
	Mineral	Iodine (μg), sodium (mg), calcium (mg)	13
	Vitamin	Vitamin C (mg), gamma-tocopherol (mg), pantothenic acid (μg)	21
	Water	Water (g)	1
	Ash	Ash (g)	1
Ingredients ^†^	Cereals	Brown rice, rice cake (mochi), pasta	76
	Potatoes and starches	Sweet potato, potato, starch	29
	Sugars and sweeteners	Superfine sugar, honey, brown sugar	16
	Pulses	Green beans, green beans, soy beans	42
	Nuts and seeds	Walnuts, sesame, peanuts	25
	Vegetables	Purple onion, parsley, cabbage	186
	Fruits	Apple, banana, strawberry	78
	Mushrooms	Shiitake mushroom, enoki mushroom, eryngii mushroom	20
	Algae	Edible brown algae (hijiki), kelp, Wakame seaweed	30
	Fish, mollusks, and crustaceans	Horse mackerel, mackerel, shrimp	157
	Meat	Pork, beef, chicken	92
	Eggs	Chicken eggs, quail eggs, silky eggs	10
	Milk and dairy products	Milk, yogurt, cheese	28
	Fats and oils	Olive oil, sesame oil, rapeseed oil	14
	Confectionaries	Donuts, jelly, cookies	11
	Beverages	Rice wine, whiskey, coffee	31
	Seasonings and spices	Pepper, mirin, doubanjiang, oyster sauce, chicken broth	100
	Prepared foods	Gyoza (frozen), fried squid (for frying, frozen), curry (beef, retort pouch)	8
	Original ingredients	MCT oil, bonito flake, protein powder	178

^†^ Nutritional and ingredient data were referenced using the Standard Tables of Food Composition in Japan 2015 (Seventh Revised Edition).

**Table 2:** Assessments of the six machine learning models in terms of predicting dietary styles.

Dietary Style	Models	Accuracy	AUC	F1-Score	MCC
Japanese diet	RFC	0.86	0.93	0.86	0.71
	LR	0.86	0.93	0.86	0.71
	SVC	0.86	0.93	0.86	0.72
	XGB	0.88	0.94	0.88	0.75
	LGBM	0.88	0.94	0.88	0.76
	DNN	0.86	0.94	0.86	0.72
Chinese diet	RFC	0.95	0.95	0.84	0.68
	LR	0.91	0.93	0.79	0.61
	SVC	0.93	0.93	0.81	0.63
	XGB	0.94	0.95	0.83	0.66
	LGBM	0.93	0.96	0.83	0.67
	DNN	0.89	0.91	0.77	0.56
Western diet	RFC	0.88	0.95	0.87	0.75
	LR	0.89	0.95	0.88	0.77
	SVC	0.89	0.95	0.88	0.77
	XGB	0.89	0.96	0.88	0.77
	LGBM	0.89	0.96	0.88	0.76
	DNN	0.90	0.95	0.89	0.78

AUC, area under the curve; DNN, deep neural network; LGBM, light gradient boosting machine; LR, logistic regression; MCC, Matthew’s correlation coefficient; RFC, random forest classifier; SVC, support vector classifier; XGB, extreme gradient boosting.

**Table 3:** Top 10 among the 604 features in the six machine learning models in the Japanese dietary style.

RFC		LR		SVC		XGB		LGBM ^a^		DNN	
Features	+/− ^b^	Features	+/−	Features	+/−	Features	+/−	Features	+/−	Features	+/−
Include dairy products	−	Soy sauce taste ^‡^	+	Anytime	N.A.	Soy sauce taste ^‡^	+	Soy sauce taste ^‡^	+	Soy sauce taste ^‡^	+
Soy sauce taste ^‡^	+	Chicken broth	−	Chicken broth		Olive oil	−	Olive oil	−	Chicken broth	−
Olive oil	−	Consommé	−	Consommé		Include dairy products	−	Include dairy products	−	Sweetener (sugar, mirin, honey)	+
Iodine ^‡^	+	Olive oil	−	Korean food		Miso ^‡^	+	Miso ^‡^	+	No stimulation to oral cavity	+
Mirin ^‡^	+	Dashi flavor ^‡^	+	Sauce (Worcestershire sauce)		Iodine ^‡^	+	Mirin ^‡^	+	Other seasoning	−
Chicken broth	−	Sweetener (sugar, mirin, honey)	+	Purple onion		Pepper	−	Dashi flavor ^‡^	+	Include foods with no measurement of potassium	−
Pepper	−	Include foods with no measurement of potassium	−	Oyster sauce		Dashi flavor	+	Dashi ^‡^	+	Consommé	−
Include tomato	−	Pepper	−	Pepper		Mirin ^‡^	+	Pepper	−	Soy sauce ^‡^	+
Consommé	−	Miso ^‡^	+	Ethnic food		Chicken broth	−	Include tomato	−	Miso ^‡^	+
Dashi ^‡^	+	Allergen-free of pork	+	Olive oil		No stimulation to oral cavity	+	Iodine ^‡^	+	Room temperature	+

DNN, deep neural network; LGBM, light gradient boosting machine; LR, logistic regression; N.A., not available; RFC, random forest classifier; SVC, support vector classifier; XGB, extreme gradient boosting. ^a^ The best model for the Japanese diet was the LGBM. ^b^ +: positive correlation, −: negative correlation. The correlation coefficient was analyzed using the Shapley additive explanations. ^‡^ well-predicted features.

**Table 4:** Top 10 among the 604 features in the six machine learning models in the Chinese dietary style.

RFC ^a^		LR		SVC		XGB		LGBM		DNN	
Features	+/− ^b^	Features	+/−	Features	+/−	Features	+/−	Features	+/−	Features	+/−
Sesame oil ^‡^	+	Chicken broth ^‡^	+	Chicken broth ^‡^	N.A.	Sesame oil ^‡^	+	Sesame oil ^‡^	+	Chicken broth ^‡^	+
Chicken broth ^‡^	+	Oyster sauce ^‡^	+	Oyster sauce ^‡^		Chicken broth ^‡^	+	Chicken broth ^‡^	+	Sesame oil ^‡^	+
Oyster sauce ^‡^	+	Sesame oil ^‡^	+	Sesame oil ^‡^		Oyster sauce ^‡^	+	Oyster sauce ^‡^	+	Sesame flavor ^‡^	+
Starch ^‡^	+	Allergen-free of sesame	+	Diets for morning sickness		Starch	+	Olive oil	−	Oyster sauce ^‡^	+
Gamma-tocopherol	+	Sesame flavor	+	For party		Doubanjiang ^‡^	+	Mirin	−	Allergen-free of sesame	−
Doubanjiang ^‡^	+	Include potato	+	Include green onion		Mirin	−	Iodine	−	For lunch	+
Allergen-free of sesame	−	Include green onion	+	Doubanjiang ^‡^		Allergen-free of sesame	− ^‡^	Doubanjiang ^‡^	+	Include potato	+
Fry	+	Fry	+	Sesame flavor		Include dairy products	−	Starch ^‡^	+	Side dish	−
Sodium	+	Sauce (Worcestershire sauce)	+	For breakfast		Olive oil	−	Pantothenic acid	+	Other seasoning	+
Mirin	−	Throughout a year	+	Sauce (Worcestershire sauce)		Iodine	−	Easy cooking	+	Very low fat and/or energy percent	−

DNN, deep neural network; LGBM, light gradient boosting machine; LR, logistic regression; N.A., not available; RFC, random forest classifier; SVC, support vector classifier; XGB, extreme gradient boosting. ^a^ The best model for the Chinese diet was the RFC. ^b^ +: positive correlation, −: negative correlation. The correlation coefficient was analyzed using Shapley additive explanations. ^‡^ well-predicted features.

**Table 5:** Top 10 of the 604 features in the six machine learning models in the Western dietary style.

RFC		LR		SVC		XGB		LGBM		DNN ^a^	
Features	+/− ^b^	Features	+/−	Features	+/−	Features	+/−	Features	+/−	Features	+/−
Include dairy products ^‡^	+	Olive oil ^‡^	+	Ethnic food	N.A.	Include dairy products ^‡^	+	Olive oil ^‡^	+	Olive oil ^‡^	+
Olive oil ^‡^	+	Include dairy products ^‡^	+	Olive oil ^‡^		Olive oil ^‡^	+	Include dairy products ^‡^	+	Include dairy products ^‡^	+
Soy sauce taste	−	Include tomato ^‡^	+	Consommé ^‡^		Soy sauce taste	−	Vitamin C	+	Include tomato ^‡^	+
Sesame oil	−	Consommé ^‡^	+	Within one hour cooking		Sesame oil	−	Soy sauce taste	−	Soy sauce taste	−
Milk and dairy products ^‡^	+	Soy sauce taste	−	Thicken		Vitamin C	+	Sesame oil	−	Polyunsaturated fatty acids	−
Consommé ^‡^	+	Low fat energy percent	−	Include garlic ^‡^		Consommé ^‡^	+	Consommé ^‡^	+	Allergen-free of apple	−
Soy sauce	−	Milk and dairy products ^‡^	+	Include tomato ^‡^		Include tomato ^‡^	+	Include tomato ^‡^	+	Salty	+
Gamma tocopherol	−	Ethnic food	−	Wooden pestle		Rice wine	−	Rice wine	−	Very low fat and/or energy percent	−
Rice wine	−	Include garlic ^‡^	+	Pasta		Parsley	+	Parsley	+	Include garlic ^‡^	+
Sesame flavor	−	No stimulation to oral cavity	−	Calcium fortification		Miso	−	Miso	−	Caution about germ growth	+

DNN, deep neural network; LGBM, light gradient boosting machine; LR, logistic regression; N.A., not available; RFC, random forest classifier; SVC, support vector classifier; XGB, extreme gradient boosting. ^a^ The best model for the Western diet was the DNN. ^b^ +: positive correlation, −: negative correlation. The correlation coefficient was analyzed using Shapley additive explanations. ^‡^ well-predicted features.

## Data Availability

The original contributions presented in the study are included in the article/supplementary material, further inquiries can be directed to the corresponding author.

## Supplementary Materials

The following supporting information can be downloaded at https://www.mdpi.com/article/10.3390/foods13050667/s1, Figure S1: The confusion matrix for the Japanese dietary style, Figure S2: The confusion matrix for the Chinese dietary style, Figure S3: The confusion matrix for the Western dietary style, Figure S4: ROC curves for the Japanese dietary style, Figure S5: ROC curves for the Chinese dietary style, Figure S6: ROC curves for the Western dietary style, Table S1: Cooking recipes in Japanese, Chinese, and Western dietary styles.
