# Supplementary material for: Development of a Machine Learning Model for Classifying Cooking Recipes According to Dietary Styles
Source: Foods. 2024 Feb 22;13(5):667. doi: 10.3390/foods13050667 (PMC10930448; doi:10.3390/foods13050667)
Supplement: Supplementary file 1 [file foods-13-00667-s001.zip › foods-2846762-SI.pdf]

Supplementary Materials

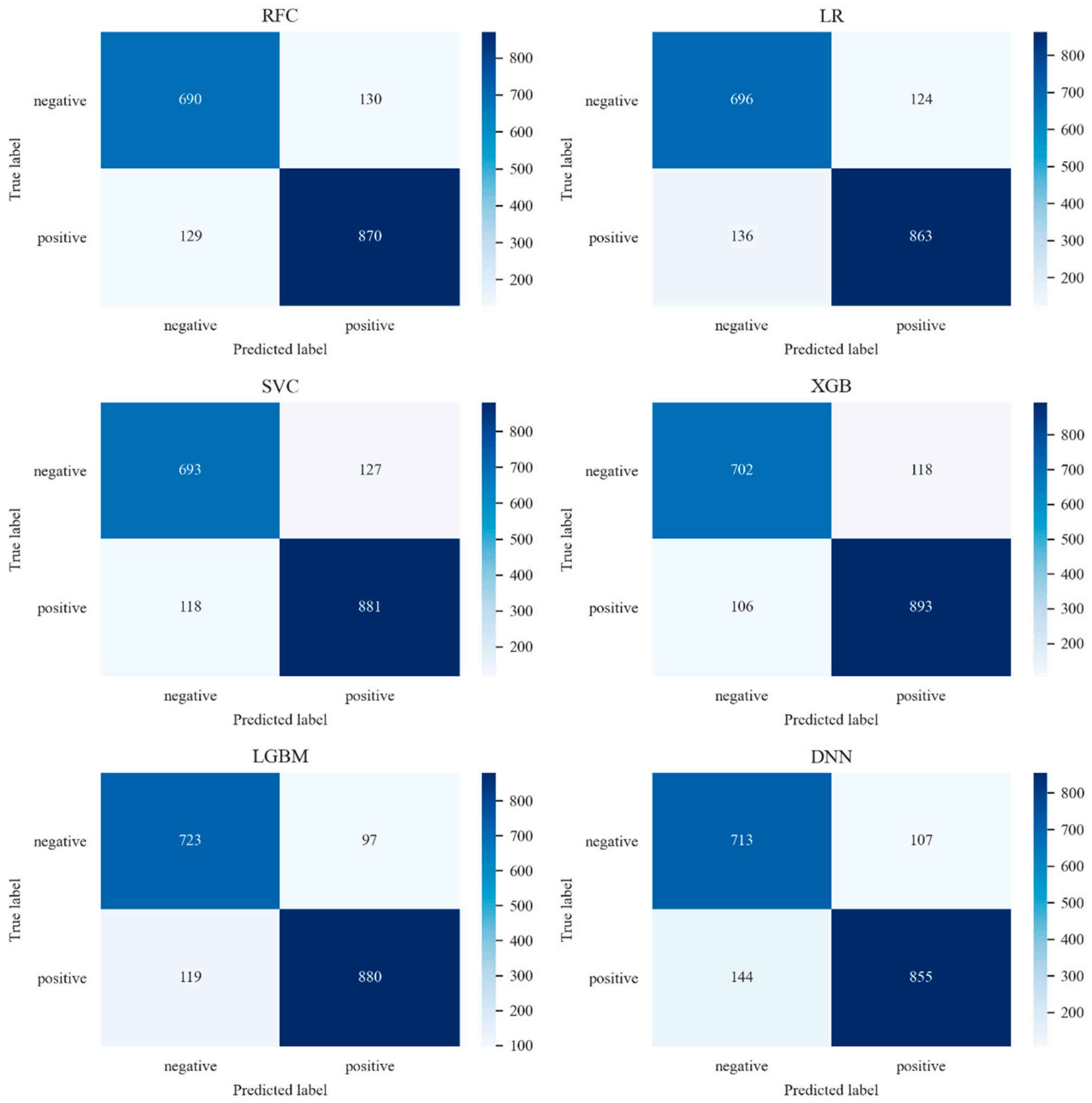

Figure S1. The confusion matrix for the Japanese dietary style.

DNN, deep neural network; LGBM, light gradient boosting machine; LR, logistic regression; RFC, random forest classifier; SVC, support vector classifier; XGB, extreme gradient boosting.

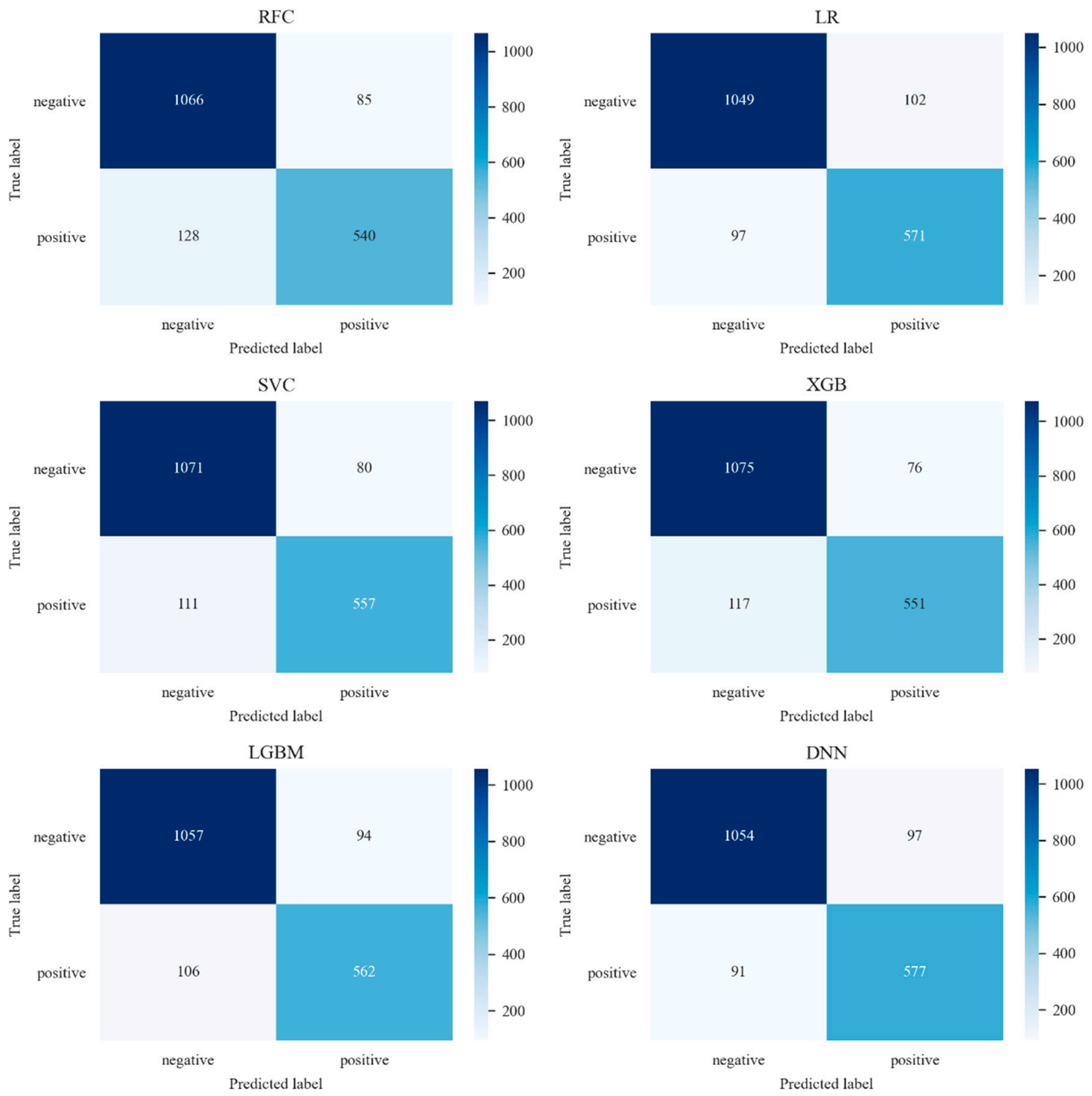

Figure S2. The confusion matrix for the Chinese dietary style.

DNN, deep neural network; LGBM, light gradient boosting machine; LR, logistic regression; RFC, random forest classifier; SVC, support vector classifier; XGB, extreme gradient boosting.

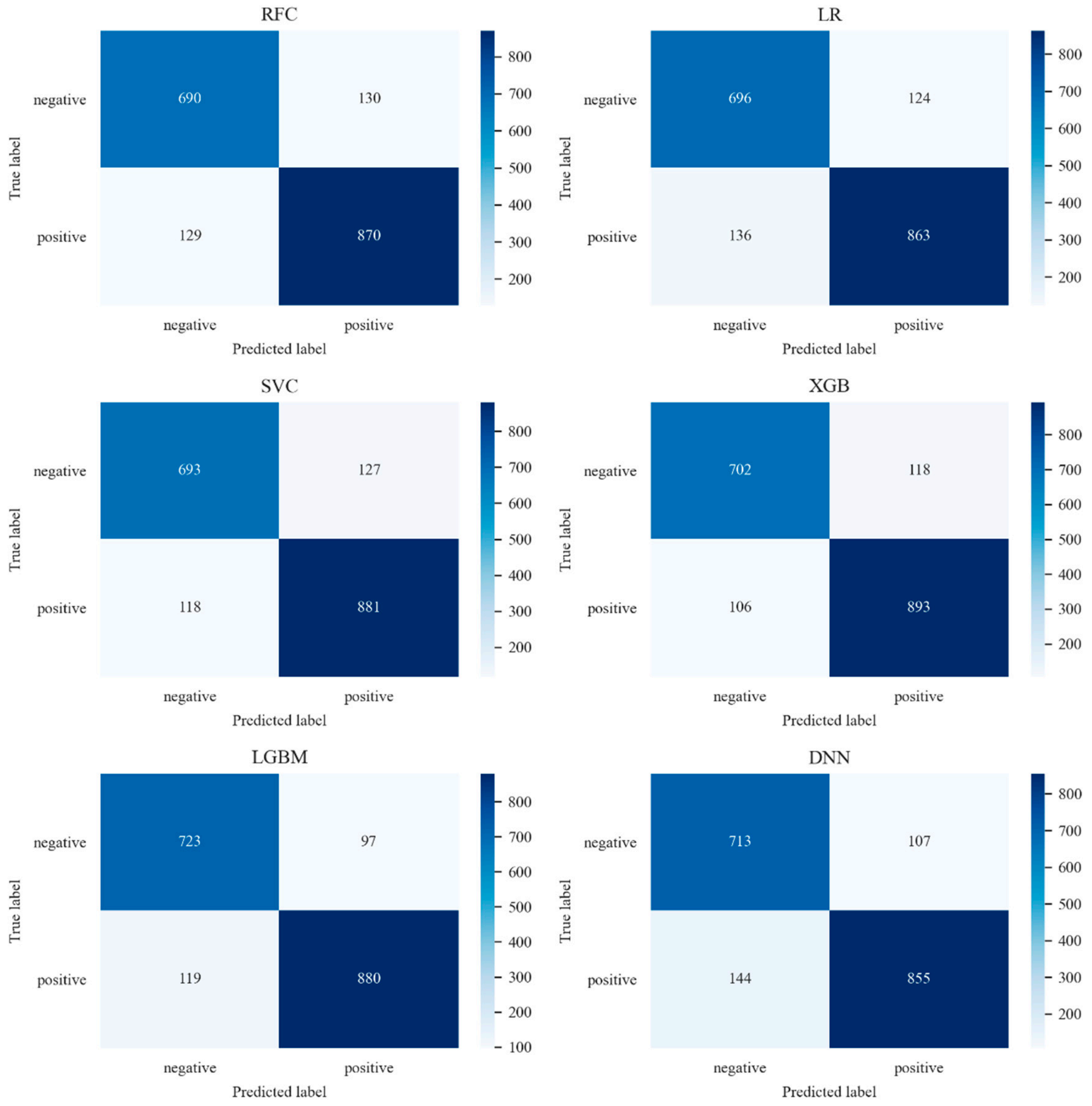

Figure S3. The confusion matrix for the Western dietary style.

DNN, deep neural network; LGBM, light gradient boosting machine; LR, logistic regression; RFC, random forest classifier; SVC, support vector classifier; XGB, extreme gradient boosting.

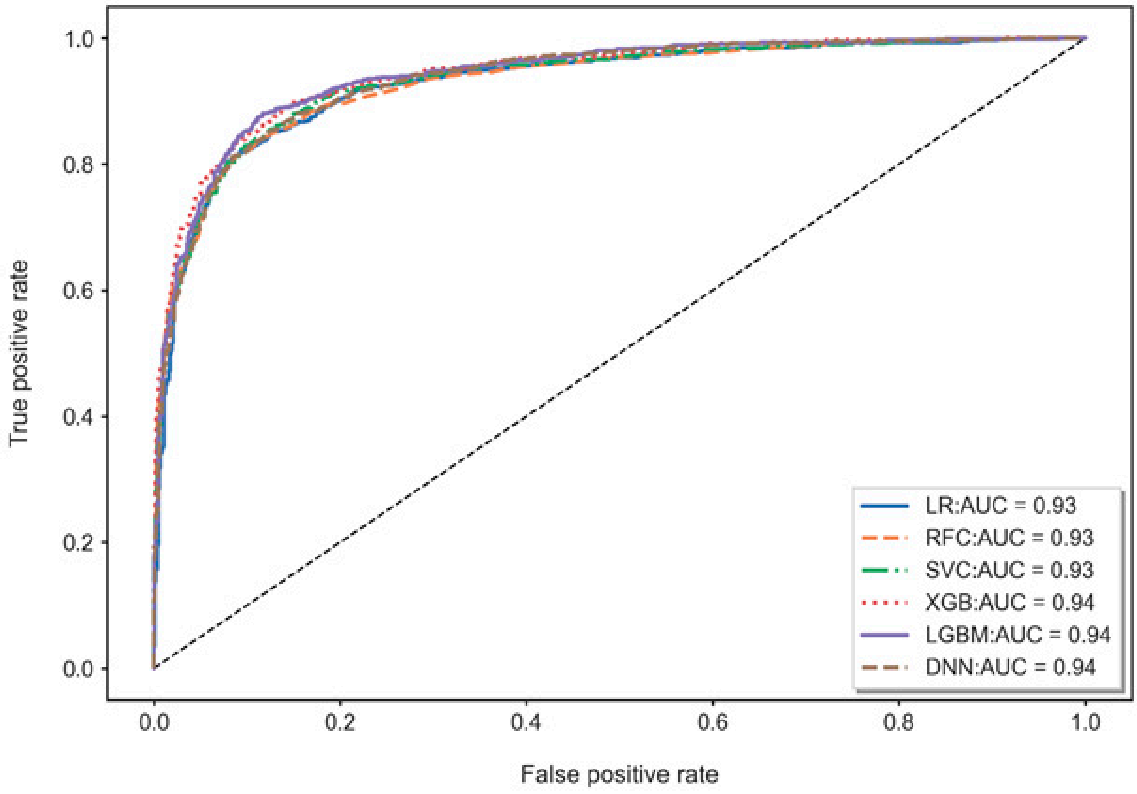

Figure S4. ROC curves for the Japanese dietary style.

AUC, area under the curve; DNN, deep neural network; LGBM, light gradient boosting machine; LR, logistic regression; MCC, Matthew's correlation coefficient; RFC, random forest classifier; SVC, support vector classifier; XGB, extreme gradient boosting.

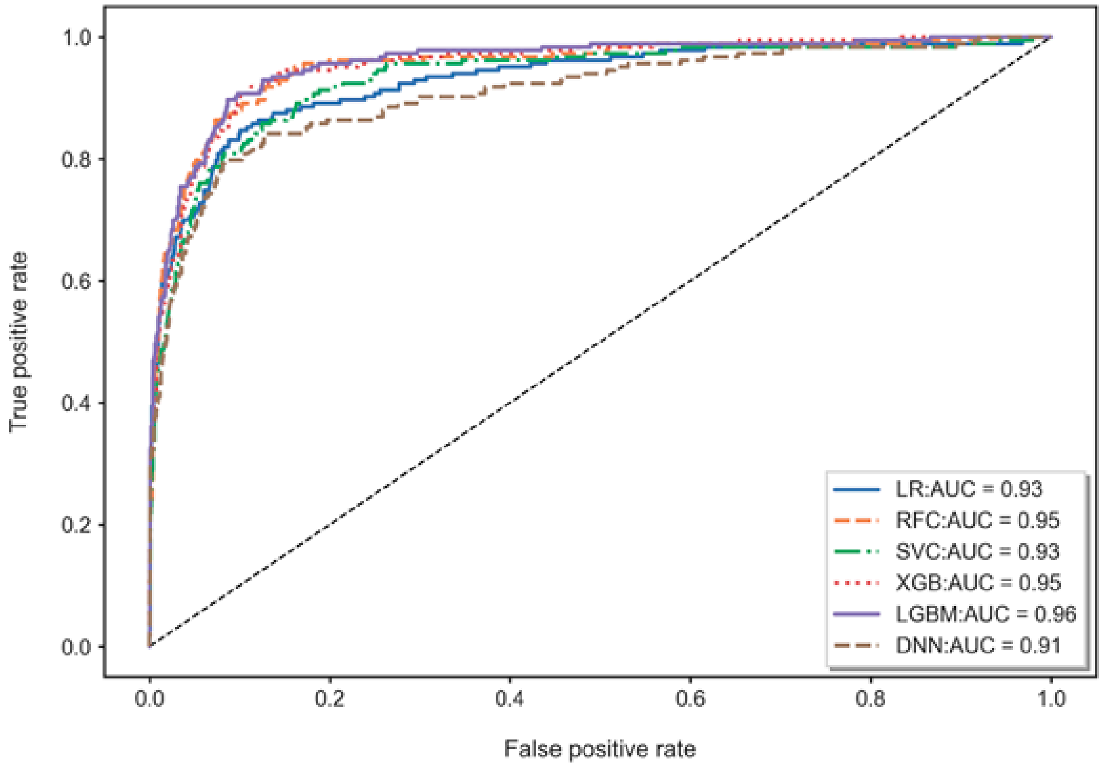

Figure S5. ROC curves for the Chinese dietary style.

AUC, area under the curve; DNN, deep neural network; LGBM, light gradient boosting machine; LR, logistic regression; MCC, Matthew's correlation coefficient; RFC, random forest classifier; SVC, support vector classifier; XGB, extreme gradient boosting.

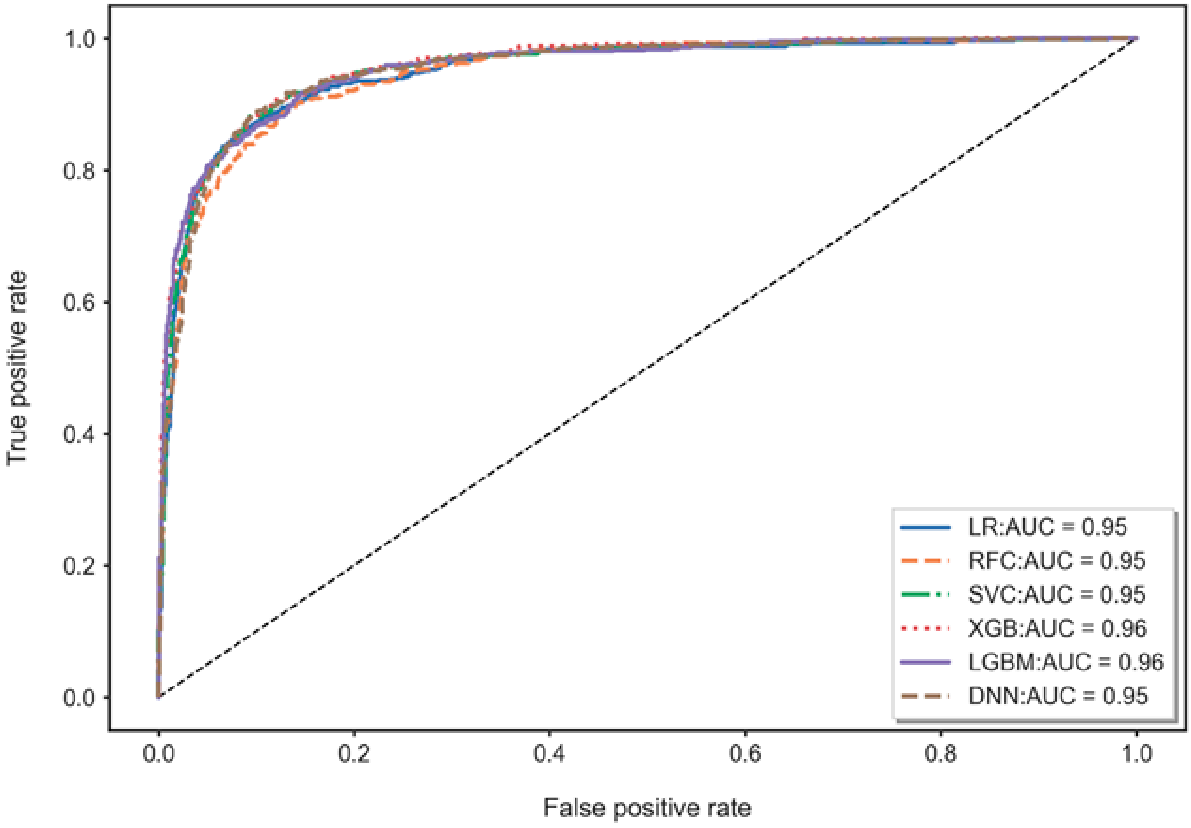

Figure S6. ROC curves for the Western dietary style.

AUC, area under the curve; DNN, deep neural network; LGBM, light gradient boosting machine; LR, logistic regression; MCC, Matthew's correlation coefficient; RFC, random forest classifier; SVC, support vector classifier; XGB, extreme gradient boosting.

Table S1. Cooking recipes in Japanese, Chinese, and Western dietary styles.

Dietary style	Recipe
Japanese	URL: https://oishi-kenko.com/recipes/16416<br>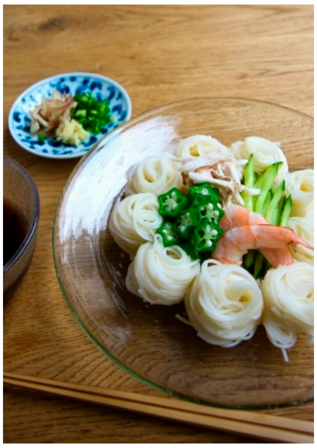<br>**Recipe name:** Chilled somen noodles with few ingredients

Ingredients and seasonings	Weight per person
Somen (dry)	75g
Shrimp (about 10g each)	20g
Chicken fillet	20g
Sake (rice wine)	1/2 teaspoon (2.5 g)
Cucumber	20g
Okra	10g
Mentsuyu (soup stock based on soy sauce with bonito and kelp soup stock)	50g
**For shrimp preparation**	1/2 teaspoon (1.5 g)
Potato starch	
Salt	0.2 g
**Condiment**	5g
Myoga	
Onion (cut into small pieces)	3g
Ginger (grated)	2g

Nutrients	Unit per person
Energy	299 kcal
Salt equivalent amount	2.2g
Protein	17.4g
Lipid	1.2g
Carbohydrates	58.7g
Sugar	55.9g
Dietary fiber	2.8g
Water soluble dietary fiber	0.8g
Insoluble dietary fiber	2.0g
Potassium	290mg
Calcium	50mg
Magnesium	42mg
Phosphorus	180mg
Iron	1.1mg
Zinc	1.0mg
Iodine	2 μg
Cholesterol	45mg
Vitamin B1	0.09mg
Vitamin B2	0.09mg
Vitamin C	6mg
Vitamin B6	0.20mg
Vitamin B12	0.4 μg
folic acid	44 μg
Vitamin A	17 μg
Vitamin D	0.0 μg
Vitamin K	20 μg
Vitamin E	0.9mg
Saturated fatty acids	0.24g
Monounsaturated fatty acids	0.14 g
Polyunsaturated fatty acids	0.47g

URL: https://oishi-kenko.com/recipes/16661

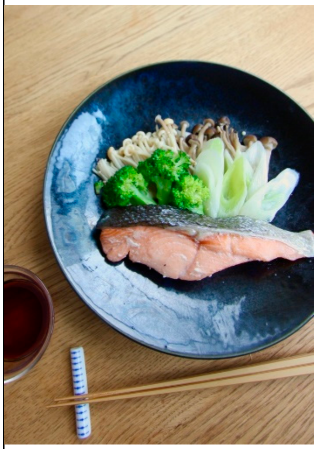

Recipe name: Microwave steamed salmon and vegetables

Ingredients and seasonings	Weight per person
Salmon (fillet)	80g
Sake (rice wine)	1 teaspoon (5 g)
Salt	0.2 g
Green onion	25g
Broccoli	20g
Shimeji mushrooms	15g
Enoki Mushroom	15g
Ponzu soy sauce (Japanese Citrus Sauce)	1 plus 2/3 teaspoons (10 g)

Nutrients	Unit per person
Energy	134kcal
Salt equivalent amount	0.9g
Protein	10.4g
Lipid	3.5g
Carbohydrates	6.3g
Sugar	3.5g
Dietary fiber	2.8g
Water soluble dietary fiber	0.4g
Insoluble dietary fiber	2.3g
Potassium	557mg
Calcium	33mg
Magnesium	39mg
Phosphorus	259mg
Iron	1.0mg
Zinc	0.8mg
Iodine	4 μg
Cholesterol	48mg
Vitamin B1	0.23mg
Vitamin B2	0.28mg
Vitamin C	35mg
Vitamin B6	0.64mg
Vitamin B12	4.7μg
folic acid	96μg
Vitamin A	26μg
Vitamin D	25.8μg
Vitamin K	44μg
Vitamin E	1.6mg
Saturated fatty acids	0.67g
Monounsaturated fatty acids	1.37g
Polyunsaturated fatty acids	0.87g

Chinese	URL: https://oishi-kenko.com/recipes/16567

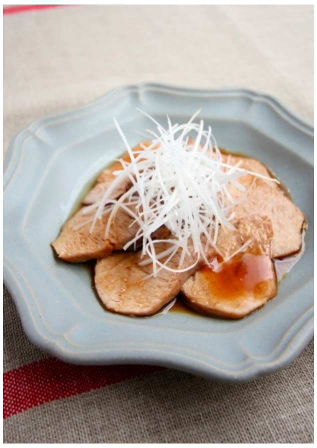

Recipe name: Braised pork

Ingredients and seasonings	Weight per person
Pork thigh chunks	80g
Green onion	10g
**Broth**	5g
Green onion (green part)	
Ginger (with skin)	2g
Garlic	1g
Sake (rice wine)	2/3 tablespoons (10 g)
Soy sauce	1 tablespoon (6 g)
Oyster sauce	2/3 teaspoon (4 g)
Sugar	1 teaspoon (3 g)
Vinegar	1/2 teaspoon (2.5 g)
Red chili pepper (optional)	a small amount
Water	75ml

Nutrients	Unit per person
Energy	175kcal
Salt equivalent amount	1.4g
Protein	17.4g
Lipid	8.2g
Carbohydrates	6.2g
Sugar	5.8g
Dietary fiber	0.4g
Water soluble dietary fiber	0.1g
Insoluble dietary fiber	0.3g
Potassium	346mg
Calcium	10mg
Magnesium	28mg
Phosphorus	180mg
Iron	0.8mg
Zinc	1.8mg
Iodine	0μg
Cholesterol	54mg
Vitamin B1	0.73mg
Vitamin B2	0.19mg
Vitamin C	2mg
Vitamin B6	0.29mg
Vitamin B12	0.3μg
folic acid	12μg
Vitamin A	5μg
Vitamin D	0.1μg
Vitamin K	2μg
Vitamin E	0.3mg
Saturated fatty acids	2.88g
Monounsaturated fatty acids	3.39g
Polyunsaturated fatty acids	1.00g

URL: https://oishi-kenko.com/recipes/16565

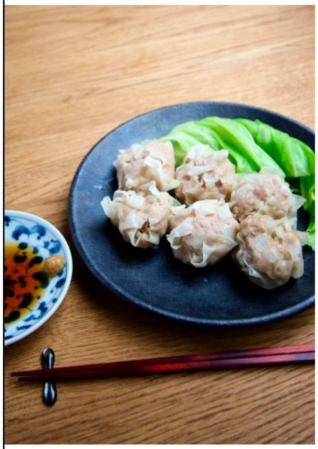

Recipe name: Easy shumai using a microwave

Ingredients and seasonings	Weight per person
Minced pork	60g
Onion	60g
Shumai skin	18g
Cabbage (or lettuce)	40g
**For seasoning**	2g
Ginger (grated)	
Sugar	2/3 teaspoon (2 g)
Soy sauce	1/3 teaspoon (2 g)
Sesame oil	1/2 teaspoon (2 g)
Potato starch	1/2 teaspoon (1.5 g)
**Sauce**	1 teaspoon (5 g)
Vinegar	
Soy sauce	1/2 teaspoon (3 g)
Mustard (optional)	1/3 teaspoon (2 g)

Nutrients	Unit per person
Energy	247kcal
Salt equivalent amount	1.0g
Protein	13.7g
Lipid	13.0g
Carbohydrates	22.4g
Sugar	20.3g
Dietary fiber	2.1g
Water soluble dietary fiber	0.5g
Insoluble dietary fiber	1.5g
Potassium	382mg
Calcium	37mg
Magnesium	32mg
Phosphorus	123mg
Iron	1.2mg
Zinc	2.0mg
Iodine	1μg
Cholesterol	45mg
Vitamin B1	0.48mg
Vitamin B2	0.17mg
Vitamin C	20mg
Vitamin B6	0.36mg
Vitamin B12	0.4μg
folic acid	40μg
Vitamin A	6μg
Vitamin D	0.2μg
Vitamin K	35μg
Vitamin E	0.4mg
Saturated fatty acids	4.13g
Monounsaturated fatty acids	5.49g
Polyunsaturated fatty acids	2.03g

URL: https://oishi-kenko.com/recipes/16486

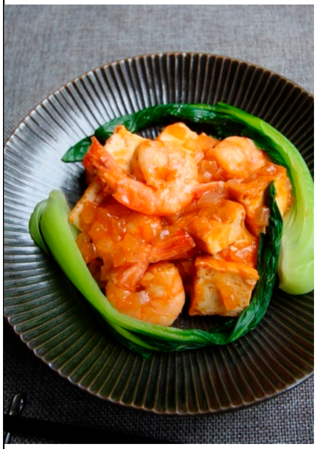

Recipe name: Stir-fried shrimp and tofu with chili sauce

Ingredients and seasonings	Weight per person
Shrimp (about 10g)	60g
Potato starch (for shrimp preparation)	as needed
Fried tofu	50g
Onion	50g
Bok-choy	20g
Salad oil	3/4 teaspoon (3g)
Garlic (chopped)	2g
**Sauce**	1 plus 2/3 teaspoons (1
Ketchup	0g)
Sugar	1 teaspoon (3g)
Soy sauce	1/2 teaspoon (3g)
Bean sauce	1/3 teaspoon (2g)
Chicken soup base	1/6 teaspoon (0.5)
Water	60ml
**Potato starch with water**	1/3 teaspoon (1g)
Potato starch	
Water	1/2 teaspoon (3g)

Nutrients	Unit per person
Energy	198kcal
Salt equivalent amount	1.6g
Protein	18.4g
Lipid	9.2g
Carbohydrates	13.2g
Sugar	11.4g
Dietary fiber	1.8g
Water soluble dietary fiber	0.6g
Insoluble dietary fiber	1.1g
Potassium	418mg
Calcium	193mg
Magnesium	61mg
Phosphorus	242mg
Iron	2.7mg
Zinc	1.5mg
Iodine	7μg
Cholesterol	97mg
Vitamin B1	0.09mg
Vitamin B2	0.07mg
Vitamin C	10mg
Vitamin B6	0.26mg
Vitamin B12	0.7μg
folic acid	63μg
Vitamin A	41μg
Vitamin D	0.0μg
Vitamin K	35μg
Vitamin E	2.2mg
Saturated fatty acids	1.22g
Monounsaturated fatty acids	2.81g
Polyunsaturated fatty acids	4.13g

Western	URL: https://oishi-kenko.com/recipes/16644

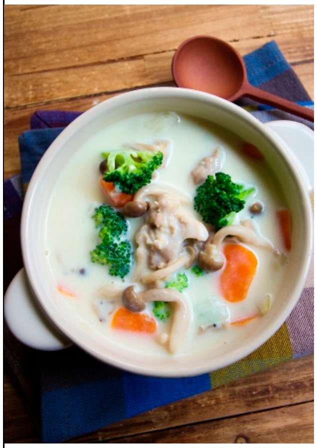

Recipe name: White stew with mushrooms

**Ingredients and seasonings**	**Weight per person**
Chicken thigh (without skin)	60g
Onion	50g
Carrot	20g
Shimeji mushrooms	40g
Broccoli	30g
Butter	1/2 tablespoon (6 g)
Flour	2/3 tablespoon (6 g)
Consommé	1/2 teaspoon (1.5 g)
Water	50ml
Milk	100ml
Salt	0.5 g
Pepper	A small amount

Nutrients	Unit per person
Energy	238kcal
Salt equivalent amount	1.5g
Protein	18.7g
Lipid	12.3g
Carbohydrates	19.9g
Sugar	15.5g
Dietary fiber	4.4g
Water soluble dietary fiber	0.9g
Insoluble dietary fiber	3.3g
Potassium	775mg
Calcium	144mg
Magnesium	45mg
Phosphorus	305mg
Iron	1.2mg
Zinc	2.1mg
Iodine	17μg
Cholesterol	77mg
Vitamin B1	0.26mg
Vitamin B2	0.42mg
Vitamin C	49mg
Vitamin B6	0.44mg
Vitamin B12	0.5μg
folic acid	101μg
Vitamin A	227 μg
Vitamin D	0.7μg
Vitamin K	81μg
Vitamin E	1.6mg
Saturated fatty acids	6.29g
Monounsaturated fatty acids	3.24g
Polyunsaturated fatty acids	0.84g

URL: https://oishi-kenko.com/recipes/16636

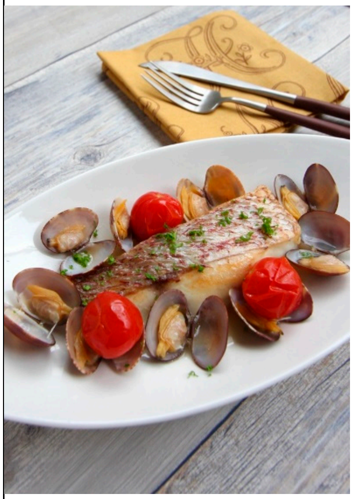

Recipe name: Sea bream and clam aqua pazza

Ingredients and seasonings	Weight per person
Sea bream (fillet)	80g
Salt	0.5g
Pepper	A small amount
Clams (60g per person with shell)	24g
Olive oil	1/2 teaspoon (2g)
Garlic	2g
Mini Tomato	30g
White wine	1 tablespoon (15g)
Water	1 tablespoon (15g)
Parsley (chopped)	A small amount

Nutrients	Unit per person
Energy	176kcal
Salt equivalent amount	1.1g
Protein	18.6g
Lipid	9.7g
Carbohydrates	3.2g
Sugar	2.6g
Dietary fiber	0.6g
Water soluble dietary fiber	0.2g
Insoluble dietary fiber	0.3g
Potassium	502mg
Calcium	31mg
Magnesium	53mg
Phosphorus	226mg
Iron	0.9mg
Zinc	0.7mg
Iodine	19μg
Cholesterol	63mg
Vitamin B1	0.28mg
Vitamin B2	0.12mg
Vitamin C	13mg
Vitamin B6	0.39mg
Vitamin B12	12.0μg
folic acid	18μg
Vitamin A	35μg
Vitamin D	5.6μg
Vitamin K	4μg
Vitamin E	2.4mg
Saturated fatty acids	2.10g
Monounsaturated fatty acids	3.67g
Polyunsaturated fatty acids	2.13g

URL: https://oishi-kenko.com/recipes/16557

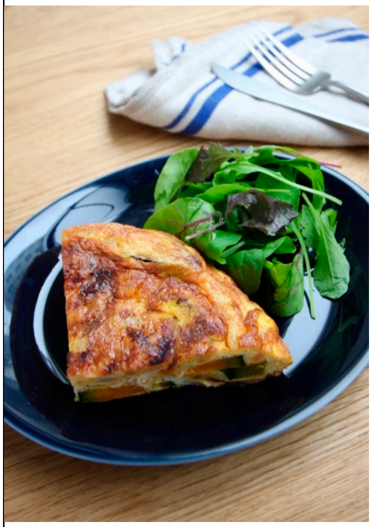

Recipe name: Spanish omelet with pumpkin

Ingredients and seasonings	Weight per person
Egg	200g
Pumpkin (with skin)	200g
Onion	120g
Olive oil	2/3 tablespoon (8g)
Baby leaf	20g
Boiled tuna	80g
Powdered cheese	1 plus 1/3 tablespoons (8g)
Salt	1/3 teaspoon (1.6g)
Pepper	0.2g

Nutrients	Unit per person
Energy	162kcal
Salt equivalent amount	0.8g
Protein	11.6g
Lipid	8.0g
Carbohydrates	13.3g
Sugar	11.0g
Dietary fiber	2.3g
Water soluble dietary fiber	0.6g
Insoluble dietary fiber	1.6g
Potassium	374mg
Calcium	66mg
Magnesium	27mg
Phosphorus	167mg
Iron	1.2mg
Zinc	1.0mg
Iodine	17μg
Cholesterol	194mg
Vitamin B1	0.08mg
Vitamin B2	0.25mg
Vitamin C	24mg
Vitamin B6	0.26mg
Vitamin B12	0.8μg
folic acid	51μg
Vitamin A	217μg
Vitamin D	2.5μg
Vitamin K	26μg
Vitamin E	2.8mg
Saturated fatty acids	2.25g
Monounsaturated fatty acids	3.84g
Polyunsaturated fatty acids	0.95g

The recipes are described in Japanese. Accessed Dec, 2023.
